# Long-term correlation of the electrocorticogram as a bioindicator of brain exposure to ionizing radiation

**DOI:** 10.1590/1414-431X20154473

**Published:** 2015-06-12

**Authors:** L.A.A. Aguiar, I.M.S. Silva, T.S. Fernandes, R.A. Nogueira

**Affiliations:** 1 Laboratório de Biofísica Teórico-Experimental e Computacional, Departamento de Morfologia e Fisiologia Animal, Universidade Federal Rural de Pernambuco, Recife, PE, Brasil; 2 Departamento de Biofísica e Radiobiologia, Universidade Federal de Pernambuco, Recife, PE, Brasil

**Keywords:** Power spectrum, Detrended fluctuation analysis, Electrocorticogram, Brain irradiation, Ionizing radiation

## Abstract

Understanding the effects of radiation and its possible influence on the nervous system are of great clinical interest. However, there have been few electrophysiological studies on brain activity after exposure to ionizing radiation (IR). A new methodological approach regarding the assessment of the possible effects of IR on brain activity is the use of linear and nonlinear mathematical methods in the analysis of complex time series, such as brain oscillations measured using the electrocorticogram (ECoG). The objective of this study was to use linear and nonlinear mathematical methods as biomarkers of gamma radiation regarding cortical electrical activity. Adult Wistar rats were divided into 3 groups: 1 control and 2 irradiated groups, evaluated at 24 h (IR24) and 90 days (IR90) after exposure to 18 Gy of gamma radiation from a cobalt-60 radiotherapy source. The ECoG was analyzed using power spectrum methods for the calculation of the power of delta, theta, alpha and beta rhythms and by means of the α-exponent of the detrended fluctuation analysis (DFA). Using both mathematical methods it was possible to identify changes in the ECoG, and to identify significant changes in the pattern of the recording at 24 h after irradiation. Some of these changes were persistent at 90 days after exposure to IR. In particular, the theta wave using the two methods showed higher sensitivity than other waves, suggesting that it is a possible biomarker of exposure to IR.

## Introduction

The possible effects of ionizing radiation (IR) on the nervous system are of great clinical interest, because this technology is widely applied in brain imaging as well as in the treatment of brain tumors (1). However, the available data on structural damage to the brain and to neurophysiological functions caused by IR and the repercussions for animal behavior are not yet conclusive (2). After accidental exposure to IR in humans, as in the case of Chernobyl, an increased incidence of schizophrenia was identified. Hypotheses suggest that IR can be a trigger for people who have a predisposition to schizophrenia, or can even cause schizophrenia (3). However, the neurophysiological basis for this is poorly understood (1).

Since the first radiobiological experiments, the sensitivity of the human brain to radiation has been a point of discussion (4). Evidence has accumulated indicating significant neurophysiological alterations as a result of exposure to ionizing radiation, such as changes in electroencephalogram (EEG) patterns, the presence of epileptiform waves and neuropsychiatric disorders (5,6).

The law of Bergoniéand Tribondeau (7) determines which cells are most (dividing and undifferentiated) and least (non-dividing and differentiated) sensitive to IR. Nerve tissue is constituted of differentiated cells; these cells are non-dividing and in the G0 stage of the cell cycle. Together with muscle cells these differentiated cells are among the most radioresistant in the human body, while the most sensitive cells are spermatogonia and erythroblasts, epidermal stem cells and gastrointestinal stem cells. After irradiation a reduction in neurogenesis was noted in the hippocampus as a result of cellular death by apoptosis, showing that these cells are highly sensitive to IR (8). Another study, involving the proliferative zone of the developing rat retina, has also shown that gamma radiation induces apoptosis (9).

The brain is a highly complex and nonlinear network (10). The most simple and usual record of its activity in routine clinical practice is obtained using an EEG, which is an important tool for understanding the behavior of the brain in normal and disease states (11). Berger (12) showed the existence of a relationship between brain states and a specific range of frequencies of the EEG. In addition, some research has associated different behaviors with specific EEG frequency bands, for example, the frequency range of 8-13 Hz is associated with quiet wakefulness and alterations of this rhythm can be associated with pathophysiological processes (13).

To identify and quantify the different brain oscillations, the spectral decomposition of this signal should be realized. A linear method used in this decomposition is the Fourier transform (FT). This method allows the characterization of the EEG through its component frequencies (11). Many studies have shown that nonlinear analysis methods, when applied to the EEG, were also efficient in identifying pathological processes (14,15). Sokunbi et al. (16), using the sample entropy and Hurst exponent methods, analyzed the brain functional magnetic resonance imaging signals in individual schizophrenics and observed a higher complexity than in healthy individuals. Some non-linear methods have been used for seizure prediction, such as the Lyapunov exponent, similarity index and correlation dimension (17). Lempel-Ziv complexity applied in brain magnetoencephalograms has been reported to have good accuracy in identifying Alzheimer disease (18) and for the identification of sedation in rats (19). Manilo and Volkova, using the approximated entropy, were able to quantify deep anesthesia (20). One important problem in analyzing the EEG is that most methods designed for stationary time series, i.e. having statistical properties such as mean and variance that do not vary with time (21), are inadequate for the analysis of non-stationary time series such as the EEG. An efficient approach for handling non-stationary series is the detrended fluctuation analysis (DFA), which has been widely used to describe the long-range temporal correlation (LRTC) in the EEG.

The DFA is one of the most widely used methods for the identification of LRTC in time series especially in being able to quantify scales in non-stationary series such as the EEG (22). Studies have successfully used it to identify changes in EEG patterns regarding neurodegenerative diseases such as Alzheimer disease (15). Other authors have identified long-range temporal correlations in the EEG of humans and characterized the exponent α-DFA to detect diseases such as depression and epilepsy (23). LRTC are also present in the hippocampus of epileptic (21) and schizophrenic patients (24). Abasolo et al. (14) evaluated the combination of spectral analysis with DFA. They suggested that the application of the LRTC regarding the investigation of the amplitude envelopes of the oscillations is more feasible in identifying degenerative changes through the EEG, such as in the Alzheimer disease model. Furthermore, the sensitivity of the α-DFA exponents in identifying changes in the EEG profile varies according to the frequency of the wave and the region recorded (24,25).

Consequently, the brain has been characterized as a sensitive tissue to ionizing radiation and could be used as a biomarker of exposure. Perhaps it could be a faster method for evaluating human acute and recent (3 months) exposures when compared with the traditional well-established method of cytogenetic dosimetry, which is based on the quantification of chromosome aberrations in peripheral lymphocytes, and is also a laborious and time consuming method of biodosimetry, despite its good specificity. The objective of the present study was to use the power spectrum and DFA to identify possible variations in the cortical electrical activity of rats after gamma-ray exposure, and attempt to identify new biomarkers of brain exposure to IR based on the methods proposed here.

## Material and Methods

### Animals and experimental design

Fifteen adult male Wistar rats (*Rattus norvegicus*) were obtained from the vivarium of the Departamento de Morfologia e Fisiologia Animal, Universidade Federal Rural de Pernambuco (UFRPE). The animals were housed using a 12-h light/dark cycle and had free access to food and water. The experimental protocol was approved by the Committee on Animal Research and Ethics of the UFRPE (#010/2012), according to the basic principles for research using animals. The animals were divided into three groups: a control (n=5) and two irradiated groups; one was evaluated at 24 h (IR24; n=5) and the other at 90 days (IR90; n=5) after exposure to IR.

### Radiation exposure

The irradiation was carried out with the animals anesthetized with 10 mg/kg xylazine and 75 mg/kg ketamine administered intraperitoneally. The anesthetized animals were irradiated at the Instituto de Radioterapia Waldemir Miranda. The absorbed dose using ^60^Co gamma radiation was 9 Gy to the top of the head followed by 9 Gy to the bottom of the head, for a total dose of 18 Gy. The gamma source was one that is used for patient radiotherapy, with a physical half-life of 5.3 years and a gamma ray range of 1.17 to 1.33 MeV. The focal length was 80 cm from the radiation source. The dose rate of the source was 123.4 cGy/min and the exposure time was 15.27 min.

### Electrocorticogram recording

The electrocorticogram (ECoG) is a methodology used to record the electrical activity of the cerebral cortex by introducing electrodes directly onto the exposed surface of the cortex. ECoG has higher resolution than EEG because of the attenuation of postsynaptic potentials by the skull that has a low conductance (26).

To record the ECoG, animals were anesthetized with 10 mg/kg xylazine and 75 mg/kg ketamine administered intraperitoneally. Rectal temperature was maintained at around 37.5±1°C with an electric heater placed under the animal. Thereafter, the head was fixed to a stereotactic base in which an incision on the top of the head exposed the periosteum, which was subsequently removed. Then, a circular hole of approximately 3 mm in diameter was trepanned on to the left hemisphere of the parietal bone, exposing the cortex. In this hole, a single Ag-AgCl electrode was placed on the cerebral cortex and an identical electrode was placed on the nasal bone to perform the ECoG.

**Figure 1:**
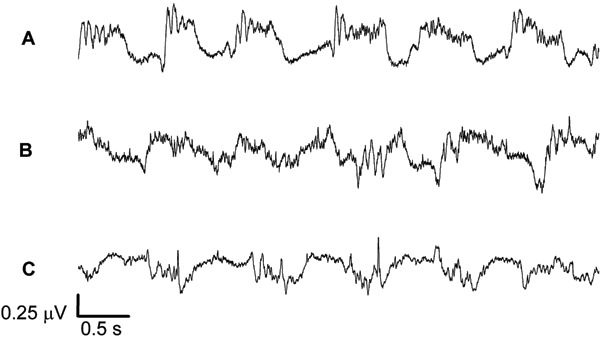
Electrocorticogram segment of 5 s duration for the following groups: control (*A*); IR24 (irradiation evaluated at 24 h) (*B*) and IR90 (irradiation evaluated at 90 days) (*C*).

Twenty minutes after the anesthesia the ECoG was registered (over a period of 30 min) using an EMG device 410C (EMG Systems, Brazil) and a sampling rate of 750 Hz. Figure 1 shows the ECoG recording for the control, IR24 and IR90 groups. After the ECoG recording, the animals were euthanized using deep anesthesia.

### Signal processing

The ECoG recordings were segmented using windows of 2 min in duration. These ECoG segments were used for power spectrum calculus to approximate to a stationary condition. The segments were imported into OriginPro 9.0 (OriginLab, USA) and filtered using a bandpass filter of the fast FT type. Then, the delta (0-4 Hz), theta (4-8 Hz), alpha (8-16 Hz), beta (16-32 Hz) and envelopes of the frequency intervals corresponding rhythms were obtained using the Hilbert transform.

The FT square of the ECoG originates its power spectrum. The mean power obtained in the power spectrum allows the estimation of the contribution of the different brain rhythms in the ECoG signal. Formally, the power spectrum for an ECoG record can be calculated as follows (Equation 1):

(Eq. 1) $${\bar{E}}_{\omega }=\frac{\int_{v_{s}}^{v_{e}}{\left|f(v)\right|}^{2}dv}{\int_{v_{s}}^{v_{e}}dv}$$

where *f*(*v*) is the FT of the*f*(*t*) signal, here represented by the ECoG. The ${\bar{E}}_{\omega }$ power spectrum normalized by a determined frequency interval $\omega =[v_{s},v_{e}]$, here represented by the different rhythms.

The calculus of the mean power for each rhythm was obtained using a routine based on Welch's method (27) and implemented in the software MATLAB (Mathworks, USA).

The DFA is a non-linear method based on fluctuation analysis of the data after removal of trends in an integrated time series (28).

The procedure to obtain the integrated time series is shown in Equation 2:

(Eq. 2) $$y(k)=\sum_{i=1}^{k}(y(i)-M)$$

where *M* is the mean value of *y (i)* with *i* = 1, 2,...,*N*.*N* is the last value of the series and*k* is an integer number that represents the superior limit of integration.

The integration above transforms the original series into an unbounded process. The series *y (k)* is divided into intervals of length n. Each interval is set by using polynomial functions, representing the trend in each interval. The function that characterizes the length of the fluctuations for a length n of the intervals used to remove the trend is shown by Equation 3:

(Eq. 3) $$F(n)=\sqrt{\frac{1}{N}\sum_{k=1}^{N}{\left[y(k)-y_{n}(k)\right]}^{2}}$$

The calculation is repeated at various interval lengths n to determine the relationship between fluctuations *F(n)* and the length of interval n. For fractal processes (self-similar), *F(n)*increases with n by the power law, as shown in Equation 4:

(Eq. 4) $$F(n)\approx n^{\alpha }$$

The self-similarity exponent α can be calculated by using the slope obtained by linear regression of graph log *F(n)* versus log n.

If α=0.5, the series was the result of a random event; α>0.5 indicated the persistent long-range correlations. The other values were: α=1 corresponding to 1/f noise (very rough landscape); the α≥1: correlations existed but ceased to be of a power-law form or a random walk-like fluctuation; and finally, α=1.5: brown noise; the integration of white noise (very smooth landscape) (28).

### Statistical analysis

All statistical data are reported as the median and interquartile range. The non-parametric Kruskal-Wallis test for significance was used; for comparison between samples, the Dunn's *post hoc* was used when required. A P value <0.05 was considered to be statistically significant.

**Figure 2:**
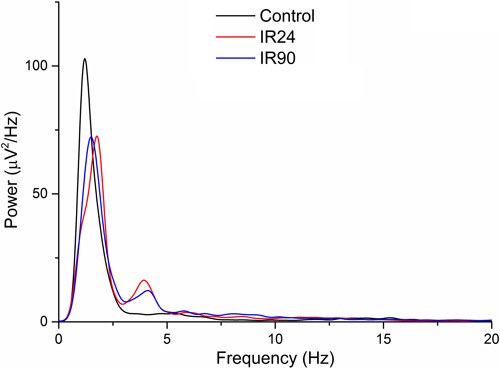
Power spectrum of the electrocorticogram for the following groups: control (black), irradiation evaluated at 24 h (IR24; red) and at 90 days (IR90; blue).

Figure 1 shows a segment of the ECoG for the control, IR24 and IR90 groups. For each experimental group, the power spectrum from each segment of the ECoG was constructed and the power calculated for different frequency intervals corresponding to the different rhythms (Figure 2).

**Figure 3:**
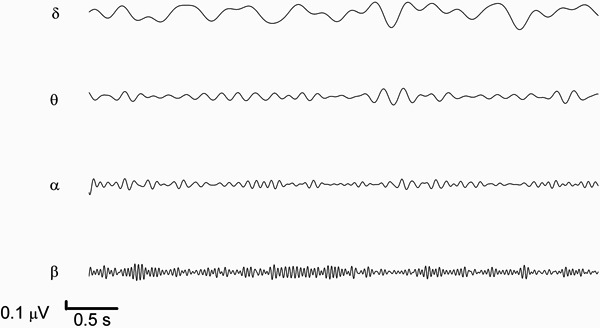
Frequency of delta (δ), theta (θ), alpha (α) and beta (β) brain rhythms filtered from the electrocorticogram.

Figure 3 shows the cortical rhythms filtered from ECoG segments using a FT filter for the control group. In this figure, changes can be noted in the delta, theta and alpha rhythms. However, the beta rhythm was not modified.

**Figure 4:**
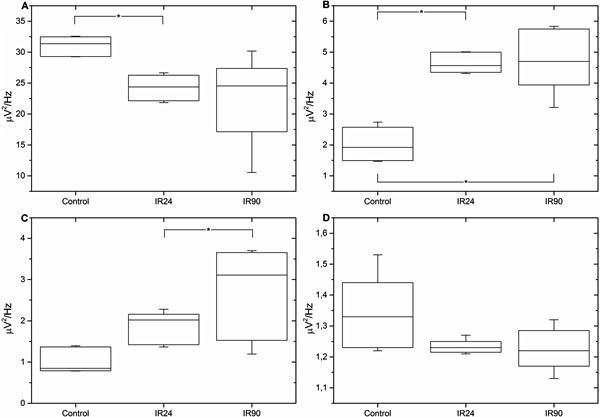
Box plot (median, 1st and 3rd quartiles, maximum and minimum) showing the results of the energy values for the groups control, irradiation evaluated at 24 h (IR24) and at 90 days (IR90) for the delta (*A*), theta (*B*), alpha (*C*) and beta (*D*) waves.*P<0.05 (Kruskal-Wallis nonparametric test).

The mean power and interquartile interval (in μV^2^/Hz) for the delta, theta, alpha and beta rhythms regarding the ECoG's can be seen in Figure 4. The IR24 and IR90 groups showed a significant reduction in the mean power for the delta rhythms relative to the control group. Theta rhythms for the two irradiated groups increased their potencies significantly compared with the control group (P<0.01). The mean powers of alpha and beta rhythms did not differ from the control. However, the mean power for the two irradiated groups differed for the alpha rhythms (P<0.05).

Mean values and the interquartile interval of the α-DFA regarding the delta, theta, alpha and beta rhythms, and the entire record of the ECoG are shown inFigure 5. The α-DFA exponent for the delta wave did not vary significantly between the groups, while for theta and beta there was only a significant difference between the control and IR24 groups. The alpha rhythm only differed significantly between the IR90 and the control groups. In the analysis of the entire record of the ECoG, the exponent of the α-DFA in both irradiated groups differed significantly from the control.

**Figure 5:**
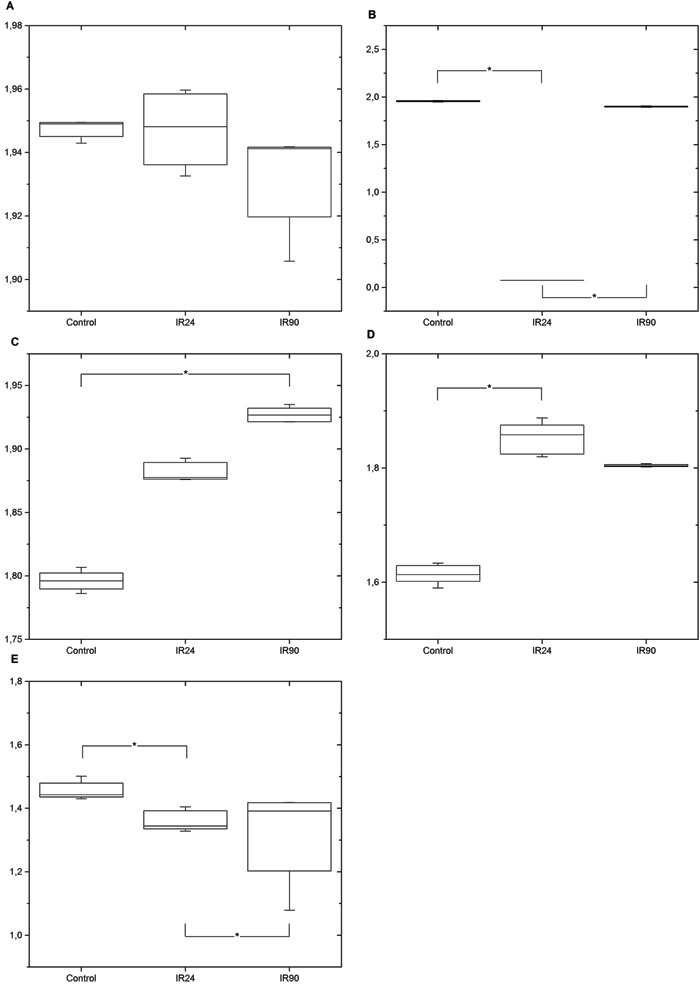
Box plot (median, 1st and 3rd quartiles, maximum and minimum) showing the results of the α-DFA for the groups control, irradiation evaluated at 24 h (IR24) and at 90 days (IR90) for the delta (*A*), theta (*B*), alpha (*C*) and beta (*D*) waves, and all electrocorticogram recordings (*E*).*P<0.05 (Kruskal-Wallis nonparametric test).

## Discussion

The focus of this study was on the investigation of the sensitivity of the power spectrum and DFA to identify changes in the ECoG profile at 24 h after IR exposure (IR24 group) and 3 months after IR exposure (group IR90). The purpose was to establish a biomarker signature of brain exposure to IR.

The power spectrum of the ECoG showed that for the IR24 group, the ECoG activity regarding theta and alpha rhythms increased relative to the control, and was consistently elevated even at 90 days after IR exposure (group IR90). The delta and beta waves were not altered by IR when compared with the control. The effects of IR on brain cortical activity in rats has not been previously reported in the literature, but Loganovsky and Yuryev (6) in a study involving survivors of Chernobyl showed that the power spectrum of the EEG increased the activities of beta and delta waves and decreased alpha and theta wave activity. Yaar et al. (29), working with humans exposed to low doses of IR (1.21-1.39 Gy), also showed an increase in beta wave activity in the power spectrum of the EEG, but no significant change was observed in other rhythms. Despite the difference between the ECoG in rats and humans, the power spectrum and the alpha coefficient of DFA can be used as a biomarker of exposure to IR, taking into consideration the specificity of each animal.

The DFA identified a reduction in the LRTC in the ECoG record, both at 24 h and 90 days after irradiation. This method when applied to the theta component of the ECoG showed that for irradiated groups the LRTC for theta rhythm activity increased significantly relative to the control group at both 24 h and 90 days after irradiation. For the alpha component, a significant increase was observed only at 90 days after irradiation when compared with the control group. For the delta and beta components of the ECoG no change was observed in the LRTC in relation to the control group.

The theta rhythm in the IR24 group was more sensitive in identifying changes in ECoG patterns. For this rhythm a correlation was shown for long-range anti-persistent rhythms and was equal to 0.07, while for the other rhythms the α-DFA coefficient was approximately 1.5, corresponding to Brownian noise. When applied to the entire ECoG, a value of α-DFA≈1.5 corresponding to Brownian noise was found. The large values of α-DFA may result from deep anesthesia (30).

In experimental animals, X-irradiation with 5 or 10 Gy reduced hippocampal neurogenesis and induced cognitive deficits at 3 months after irradiation (31), suggesting that cranial irradiation may induce hippocampal-dependent memory deficits. Active neurogenesis is responsible for learning activities, memory and spatial orientation (32) and modulation of theta rhythm (33). Therefore, changes in the theta rhythm in the present study can be related to the damage that IR induced in this theta generator; this was strongly supported by the presence of a long-range correlation in the IR24 group and an increase in the power of the theta rhythms in the IR24 and IR90 groups compared with the control group.

Computer models have shown that temporal long-range correlations are dependent on the oscillation amplitudes of the potentials generated by neural networks (34). The spread of electrical activity is dependent on the membrane system and neurotransmitters (32). Some recent studies have demonstrated that different types and sources of IR cause changes in the postsynaptic potential in the hippocampus, making this region hyperexcitable (35–37). The intracellular recordings of neurons showed that membrane properties such as resistance, the time constant, duration of the threshold of action potential and spike frequency adaptation were not significantly changed from 1 to 3 months after exposure to IR (35). Obenaus et al. (1) suggested that these neurophysiological changes have relationships with common cellular pathways, probably with an increase in excitatory neurotransmitters and a decrease in inhibitory ones.

In summary, power spectrum and detrended fluctuation analysis were sensitive in identifying changes in ECoG and showed changes in the pattern of ECoG at 24 h after irradiation; these persisted after 90 days. Applications of these methods could be used in the future to identify radiation levels as a new biomarker of brain exposure to IR.
